# Dermcidin expression in hepatic cells improves survival without *N*-glycosylation, but requires asparagine residues

**DOI:** 10.1038/sj.bjc.6603148

**Published:** 2006-05-09

**Authors:** A G Lowrie, S J Wigmore, D J Wright, I D Waddell, J A Ross

**Affiliations:** 1 Tissue Injury and Repair Group, Chancellor's Building, The University of Edinburgh Medical School, Royal Infirmary of Edinburgh, 51 Little France Crescent, Edinburgh, UK; 2 Cardiovascular and Gastrointestinal Research Department, AstraZeneca, Mereside, Alderley Park, Macclesfield SK10 4TG, UK

**Keywords:** dermcidin, liver, survival

## Abstract

Proteolysis-inducing factor, a cachexia-inducing tumour product, is an N-glycosylated peptide with homology to the unglycosylated neuronal survival peptide Y-P30 and a predicted product of the dermcidin gene, a pro-survival oncogene in breast cancer. We aimed to investigate whether dermcidin is pro-survival in liver cells, in which proteolysis-inducing factor induces catabolism, and to determine the role of potentially glycosylated asparagine residues in this function. Reverse cloning of proteolysis-inducing factor demonstrated ∼100% homology with the dermcidin cDNA. This cDNA was cloned into pcDNA3.1+ and both asparagine residues removed using site-directed mutagenesis. *In vitro* translation demonstrated signal peptide production, but no difference in molecular weight between the products of native and mutant vectors. Immunocytochemistry of HuH7 cells transiently transfected with V5-His-tagged dermcidin confirmed targeting to the secretory pathway. Stable transfection conferred protection against oxidative stress. This was abrogated by mutation of both asparagines in combination, but not by mutation of either asparagine alone. These findings suggest that dermcidin may function as an oncogene in hepatic as well as breast cells. Glycosylation does not appear to be required, but the importance of asparagine residues suggests a role for the proteolysis-inducing factor core peptide domain.

The first product of the dermcidin gene to be identified was proteolysis-inducing factor (PIF), a novel cachectic factor that was purified from a cachexia-inducing murine tumour and the urine of weight-losing patients with pancreatic cancer (Todorov *et al*, 1996; Wigmore *et al*, 2000). Screening of tissues for PIF binding demonstrated substantial binding only to skeletal muscle and liver (unpublished observation), and we have investigated the effect of PIF on liver cells. In primary hepatocytes and HepG2 cells, PIF was found to activate the transcription factors NF-*κ*B and STAT3, resulting in the stimulation of IL-8, IL-6 and CRP production, and the inhibition of transferrin production (Watchorn *et al*, 2001). Proteolysis-inducing factor was also found to induce the expression of NF-*κ*B, IL-6, IL-8, ICAM-1 and VCAM and the shedding of syndecans in the liver endothelial cell line SK-HEP-1 and human umbilical vein endothelial cells, but not in pulmonary artery endothelial cells (Watchorn *et al*, 2002). These results are consistent with a role as a cachectic factor. In addition, the roles of ICAM-1, VCAM and syndecans in development, inflammation, tissue repair and host defence (Collins *et al*, 1995; Attar *et al*, 1999; Park *et al*, 1999; Zimmermann and David, 1999) suggested for the first time that dermcidin may have a role in liver biology outwith the cachectic process.

More recently, different proteolytic fragments have been characterised, which all appear to be derived from the protein encoded by the dermcidin gene. These fragments have different roles and have been identified in different tissues. One fragment is DCD-1, a proteolytically processed 47-amino-acid C-terminus product of dermcidin secreted by eccrine sweat glands, which has antimicrobial activity against a variety of pathogenic micro-organisms (Schittek *et al*, 2001). The amino-acid sequence of DCD-1 does not overlap with that of PIF. A second product is diffusible survival evasion peptide (DSEP, or Y-P30), a neuronal survival peptide identified in the medium of oxidatively stressed neuronal cell lines (Cunningham *et al*, 1998). Y-P30 is a product of the same region of dermcidin as the core peptide of PIF (Figure 1B).

Enzymatic degradation of the PIF glycoprotein has suggested that the molecule consists of a peptide core of molecular weight 4000 Da, which is extensively *N*- and *O*-glycosylated to give an apparent mass of 24 kDa (Todorov *et al*, 1997). This may be essential to its function as a cachetic factor, as dermcidin expression does not appear sufficient to induce cachexia (Monitto *et al*, 2004). Similarly, deglycosylated PIF lacks proteolytic activity in murine gastrocnemius muscle (Todorov *et al*, 1996, 1997), and in our experiments it was the fully glycosylated PIF molecule that influenced hepatic cell NF-*κ*B and STAT3 activity and gene expression (Watchorn *et al*, 2001, 2002). However, the PIF amino-acid sequence lacks typical consensus *N*- and *O*-glycosylation sites and the mechanism of glycosylation remains unknown.

The unglycosylated PIF amino-acid sequence is predicted to be identical to that of Y-P30 (Todorov *et al*, 1996; Cunningham *et al*, 1998), and it is now believed that dermcidin may play a role in carcinogenesis via the action of this peptide. Dermcidin has been identified as a candidate oncogene in breast cancer and stable transfection has been shown to stimulate cell growth and inhibit menadione-induced cell death, as assessed by cell counting (Porter *et al*, 2003). Stable transfection of dermcidin has also been demonstrated to improve the survival of neuronal cells subjected to oxidative stress with H_2_O_2_ (Cunningham *et al*, 2002), and it is feasible that these effects are due to the activity of Y-P30. The mechanism by which this peptide improves survival appears to involve a central, calcineurin-like phosphatase domain (Cunningham *et al*, 1998). This domain contains one of the two possible *N*-glycosylation sites of PIF and lies just seven amino acids from the other.

In this study, we sought to investigate whether dermcidin expression affected the survival of hepatic cells and to examine the structural elements involved in this function. We also describe the confirmatory cloning and sequencing of the dermcidin cDNA from the PIF peptide sequence and the investigation of co-translational processing of the nascent polypeptide in an *in vitro* translation system.

## MATERIALS AND METHODS

### Computer searches

A computer search of the Lifeseq Foundation database (Incyte, Cambridge, UK) was performed to identify alignments between the 20 known N-terminal amino acids of glycosylated PIF (Todorov *et al*, 1996) and translated Incyte sequences. BLAST searches were performed using the national library of medicine facility (http://www.ncbi.nlm.nih.gov/). Post-translational modification was modelled using SignalP (http://www.cbs.dtu.dk/services /SignalP/), Net-*O*-Glyc (http://www.cbs.dtu.dk/services /NetOGlyc/) and Net-*N*-Glyc (http://www.cbs.dtu.dk/services /NetNGlyc/).

### PCR

PCR primers were designed to include the PIF/dermcidin sequence from translation initiation to the final STOP codon, clamped 5′ *Bam*H1 and Kozak sequences added to the forward primer and clamped 3′ *Eco*RI sequences added to the reverse primer (F=CTCGGATCCGCCGCCATGAGGTTCATGACTCTCC, R=CAGAATTCCTGGGTATCATTTCTCAGCT) (TAGN, Newcastle, UK). Reaction mixtures (25 *μ*l) containing 0.75 *μ*l 25 mM MgCl_2_, 2.5 *μ*l taq poly 10 × buffer, 2.5 *μ*l dNTPs, 1 mM forward and reverse primers and 1 *μ*l *taq* polymerase at a 1 in 5 dilution were made up in nuclease-free water (all reagents from Promega, Poole, UK). Proteolysis-inducing factor/dermcidin was then amplified from cDNA derived from the G361 human melanoma cell line using a standard cycle (95°C for 5 min, followed by 35 cycles of 95°C for 1 min, 56°C for 1 min and 72°C for 1 min, then 10 min at 72°C) (ECACC, Porton Down, UK). Samples were run on 1.4% agarose gels, stained with ethidium bromide and visualised under UV illumination.

### RNA preparation and reverse transcription

RNA was prepared from cells cultured in six-well plates using Trizol (Life Technologies, Paisley, UK) according to the manufacturer's instructions. Samples were DNAse treated using RQ1 DNAse (Promega) according to the manufacturer's instructions. Reverse transcription was performed in 20 *μ*l reactions containing 10 *μ*l of RNA sample plus 10 *μ*l of RT mix (4 *μ*l 25 mM MgCl_2_, 2 *μ*l reverse transcription 10 × buffer, 2 *μ*l dNTP mixture, 1 *μ*l oligo(dT)_15_ primer, 0.5 *μ*l AMV reverse transcriptase and 0.5 *μ*l recombinant RNasin ribonuclease inhibitor, all from Promega). Samples were then incubated in a thermal cycler (PHC-3, Techne) for 1 h at 42°C, followed by 5 min at 99°C.

### Cloning

Bands of the appropriate size were excised from gels and purified using a gel purification kit (Qiagen, Crawley, UK). Samples and vectors (pcDNA3.1+ and pcDNA3.1V5-His, Invitrogen) were double digested with *Bam*H1 and *Eco*R1 for 16 h at 37°C and digestion byproducts removed by gel purification. Directional ligation was performed in 10 *μ*l reactions containing 1 *μ*l T4 DNA ligase, 1 *μ*l 10 × ligation buffer (Promega), 100 ng insert and the appropriate amount of vector to give 3 : 1, 1 : 1 and 1 : 3 molar ratios of insert to vector. Ligated vectors were then transformed into TOP10 *Escherichia coli* (Invitrogen) as per the manufacturer's instructions and plated on LB agar plus 50 *μ*g ml^−1^ ampicillin (Sigma, Poole, UK). Colonies were screened for insert incorporation by PCR and incubated in 3 ml LB+50 *μ*g ml^−1^ ampicillin minibroths overnight at 37°C with agitation. Plasmids were prepared by alkaline lysis and ethanol precipitation. All plasmids were sequenced prior to large-scale culture and purification using an Endofree plasmid maxi kit (Qiagen).

### Site-directed mutagenesis

Site-directed mutagenesis was performed using a Quikchange II kit (Stratagene, Amsterdam, the Netherlands) according to the manufacturer's instructions. Primers used were: N32Q F-CCAGGATCGGGGCAGCCTTGCCATGAAGC, N32Q R- GCTTCATGGCAAGGCTGCCCCGATCCTGG, N44Q F-CAGCAGCTCAAAAGGAACAGGCAGGTGAAGACCCAG and N44Q R- CTGGGTCTTCACCTGCCTGTTCCTTTTGAGCTGCTG. Double N32Q, N44Q mutants were created by mutation of the pcDNA3.1+PIF N32Q plasmid with N44Q primers.

### *In vitro* translation

*In vitro* translation was performed using a rabbit reticulocyte system (Promega) according to the manufacturer's instructions. Radioisotopes used were ^35^S-methionine, ^14^C-leucine and ^35^S-cysteine (ICN Pharmaceuticals Ltd, Basingstoke, UK). In selected experiments, reactions were supplemented with 1.2 *μ*l canine pancreatic microsomal membranes (CPMM) (Promega). Samples were run on 10–20% tris-tricine gels (Biorad, Hemel Hempstead, UK) at 70 V on ice and visualised by fluorography.

### Transfection and cell culture

Plasmids were transfected into the HuH7 cell line (ECACC) using Fugene (Roche Applied Science, Lewes, UK) according to the manufacturer's instructions. Stable transfectants were selected by culture in 600 *μ*g ml^−1^ G418 (Sigma). Cells were maintained at 37°C in 5% CO_2_ in air atmosphere in Dulbecco's modified Eagle's medium with 10% FCS, 50 U ml^−1^ penicillin, 50 *μ*g ml^−1^ streptomycin and 2 mmol glutamine (Gibco-BRL, Paisley, UK).

### Assessment of cell survival following oxidative stress

Cells were plated in 96-well plates (Gibco) at 1.5 × 10^4^ cells per well and cultured overnight prior to the induction of oxidative stress by the addition of 75 mU ml^−1^ glucose oxidase (Sigma) for 2 h. The medium was then replaced and cells incubated under standard conditions for 4 h. Cell survival was then assessed by MTT assay. In all, 10 *μ*l of 5 mg ml^−1^ MTT was added to each well and incubated for 4 h at 37°C. Then, 100 *μ*l per well of 10% SDS, pH 3.0 was added and plates were incubated overnight at 37°C prior to reading at 570 nm on an Assayzap plate reader.

### Flow cytometry

For flow cytometry experiments cells were seeded in six-well plates at 1 × 10^6^ per well. Following glucose oxidase treatment, cells and their culture medium were harvested, pelleted and washed twice by resuspension in PBS and centrifugation at 1500 **g** for 5 min. Annexin V/PI flow cytometry was performed using a Bender Medsystems kit according to the manufacturer's instructions (Bender Medsystems, Vienna, Austria). Cleaved caspase 3 flow cytometry was performed using the cleaved caspase-3 (Asp175) (5A1) rabbit monoclonal antibody (Cell Signaling, Hitchin, UK) according to the manufacturer's instructions. Flow cytometry using the BOB78 antibody at 1 in 100 and control antibody, also of IGM isotype (Sigma), was performed as described previously (Hart *et al*, 2000). The secondary antibody used was sheep anti-mouse IgG FITC (Dako, Ely, UK). Samples were analysed using a Coulter Epics XL flow cytometer.

### Immunocytochemistry

Cells were seeded at 2 × 10^4^ cells per well in eight-well chambered slides and allowed to settle overnight prior to glucose oxidase treatment. For V5-His visualisation, cells were treated with 5 *μ*M monensin (Sigma) and visualised directly using FITC-labelled anti-V5 His antibody (Invitrogen, Paisley, UK). For assessment of cell death with annexin V, BOB78 and anti-cleaved caspase 3 cells were treated with glucose oxidase and dead cells harvested from the medium. These were then cytospun onto positively charged slides at 300 rpm for 3 min.

Annexin V staining was performed by incubation of cytospins for 5 min at room temperature in 50 *μ*l of annexin binding buffer containing 1.25 *μ*l annexin V and 1.25 *μ*l propidium iodide (Bender Medsystems).

BOB78 staining was performed by blocking for 1 h at room temperature in 20% rabbit serum, then staining for 1 h at room temperature with BOB78 diluted 1/100 in PBS. Slides were washed in PBS, then incubated at room temperature for 30 min with secondary rabbit anti-mouse FITC-conjugated immunoglobulins (Dako) diluted 1/100 in PBS. Cells were fixed in 3% paraformaldehyde for 5 min and mounted in fluorescent mounting medium (Dako) prior to visualisation on a Leica DMIRB fluorescent microscope (Wetzlar, Germany).

## RESULTS

### Cloning and structural homology of PIF

**Figure 1:**
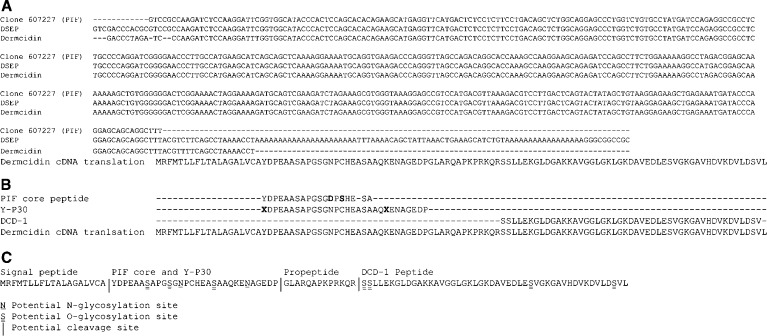
(**A**) Homology between the PIF clone 607227, DSEP and dermcidin cDNAs. Clone 607227 was identified by screening the Lifeseq Foundation database (Incyte) for sequences which, when translated, showed a high degree of homology to the PIF amino-acid sequence. A high degree of homology between the translated and untranslated sequences of the three cDNAs can also be seen to exist. The 330 bp ORF is 100% homologous in all sequences. (**B**) Amino-acid homology between PIF, Y-P30 and dermcidin. Proteolysis-inducing factor shares all but three amino acids with the predicted dermcidin protein and mismatches may be the result of sequencing artefact resulting in the conversion of cysteine to serine (Todorov *et al*, 1996). Similarly, only the two unidentifiable amino acids of Y-P30 (Cunningham *et al*, 1998) do not match with the predicted dermcidin protein. These results are subsequently consistent with both the PIF core peptide and Y-P30 arising from the dermcidin protein. The sequence of the dermcidin peptide DCD-1 does not overlap with that of Y-P30 or the PIF core peptide. (**C**) Diagram of the PIF protein arrangement showing sites of signal peptide, Y-P30 and DCD-1 cleavage. Asparagine and serine residues, which are the only potential *N*- and *O*-glycosylation sites, respectively, are underlined and can be seen to lie in the Y-P30 sequence, with the exception of the four serine residues of DCD-1. The Y-P30 and DCD-1 sequences were derived as described (Cunningham *et al*, 1998; Schittek *et al*, 2001). The signal peptide sequence and *N*- and *O*-glycosylation sites were derived by computer modelling using SignalP, Net-*N*-Glyc and Net-*O*-Glyc, respectively.

We obtained the cDNA for dermcidin through the reverse cloning of PIF. A search of the Lifeseq Foundation database identified a single clone, no. 607227, which, when translated, showed 90% similarity to the 20 N-terminal amino acids of the PIF core peptide (Figure 1A). The sequence was registered with Genbank (accession number AY590150). PCR of the coding area of this sequence allowed confirmatory cloning and sequencing from G361 cDNA. A BLAST search of the human genome database localised PIF to chromosome 12q13 and identified ∼100% homology with the dermcidin gene (AF144011) and DSEP (AY044239), the sequence encoding Y-P30. Modelling with Signal-P suggested a high likelihood that a signal peptide was cleaved between A19 and Y20 and that the protein was likely to be targeted to the secretory pathway (Figure 1C). Net-*N*-Glyc did not demonstrate typical Asn–X–Ser/Thr *N*-glycosylation sites in the PIF/dermcidin amino-acid sequence. A search of all potential *N*-glycosylation sites revealed *N*-glycosylation potentials of 0.766 and 0.6294 for N32 and N44, the former of which conforms to a previously described atypical *N*-glycosylation site (Sato *et al*, 2000). Net-*O*-Glyc modelling did not reveal any Ser/Thr sites meeting the threshold for O-glycosylation. A map of the predicted nascent polypeptide translated from the dermcidin gene is shown in Figure 1B.

### Creation and site-directed mutagenesis of expression vectors

In order to study the expression and co-translational modification of PIF/dermcidin, the full-length cDNA was directionally cloned into the mammalian expression vector pcDNA3.1+. Site-directed mutagenesis was then employed to remove the asparagine residues of the calcineurin-like phosphatase domain. The mutants created were the asparagine to glutamine substitutions N32Q and N44Q and the double asparagine to glutamine substitution N32Q N44Q. All plasmid sequences were confirmed by direct sequencing.

### *In vitro* translation of pcDNA3.1+PIF

**Figure 2:**
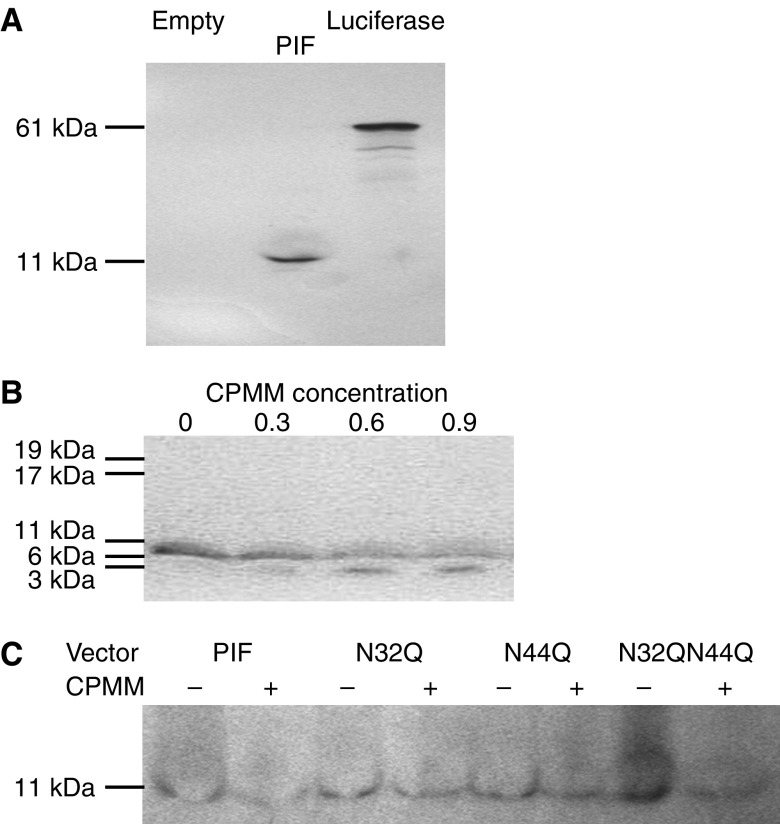
*In vitro* translation of pcDNA3.1+PIF. (**A**) Gel of ^35^S-methionine labelled *in vitro* translation reaction using the coupled T7 RNA poymerase and rabbit reticulocyte lysate system. DNA templates used were from left to right, pcDNA3.1+ (empty vector), pcDNA3.1+PIF and luciferase. Translation of PIF from the pcDNA3.1+PIF plasmid resulted in an 11 kDa product (middle lane). The 61 kDa product of the luciferase control gene can be seen in the right-hand lane. (**B**) Addition of CPMM resulted in the dose-dependent production of a 2.5 kDa band in translation reactions in which pcDNA3.1+PIF was used as a template. (**C**) Translation of the N32Q, N44Q and N32Q N44Q mutants in the presence of 1.2 *μ*l per reaction of CPMM resulted in products of molecular weight identical to the translation product of wild-type PIF/dermcidin.

To investigate co-translational processing of native and mutant DCD, *in vitro* translation using a coupled T7 RNA polymerase/rabbit reticulocyte lysate system and canine pancreatic micorsomal membranes was performed. Translation of pcDNA3.1+PIF in the presence of ^35^S-methionine gave an unprocessed product of 11 kDa (Figure 2A). Co-translational processing, in the presence of CPMM, produced a cleavage product of approximately 2.5 kDa (Figure 2B). *In vitro* translation of N32Q, N44Q and N32Q N44Q mutant vectors with ^35^S-cysteine resulted in products of molecular weight identical to wild-type PIF/dermcidin (Figure 2C).

### Targeting of PIF/dermcidin to the secretory pathway

**Figure 3:**
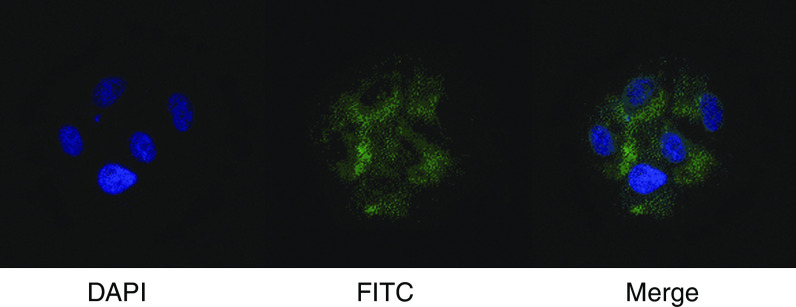
Immunocytochemistry of V5-His-tagged PIF transiently transfected into HuH7 cells in the pcDNA3.1+V5His PIF vector. Following transfection, cells were treated with the inhibitor of glycoprotein secretion monensin, fixed and co-stained with an FITC-labelled anti-V5His secondary and Hoescht 33258. FITC fluorescence was not observed without monensin treatment.

In order to study subcellular localisation, the PIF/dermcidin cDNA was cloned into the pcDNA3.1+ V5 His vector and transiently transfected into HuH7 cells. Anti-V5 His immunocytochemistry of these cells demonstrated staining only after treatment with monensin, an inhibitor of intracellular protein transport (Figure 3).

### Generation of stable transfectants

**Figure 4:**
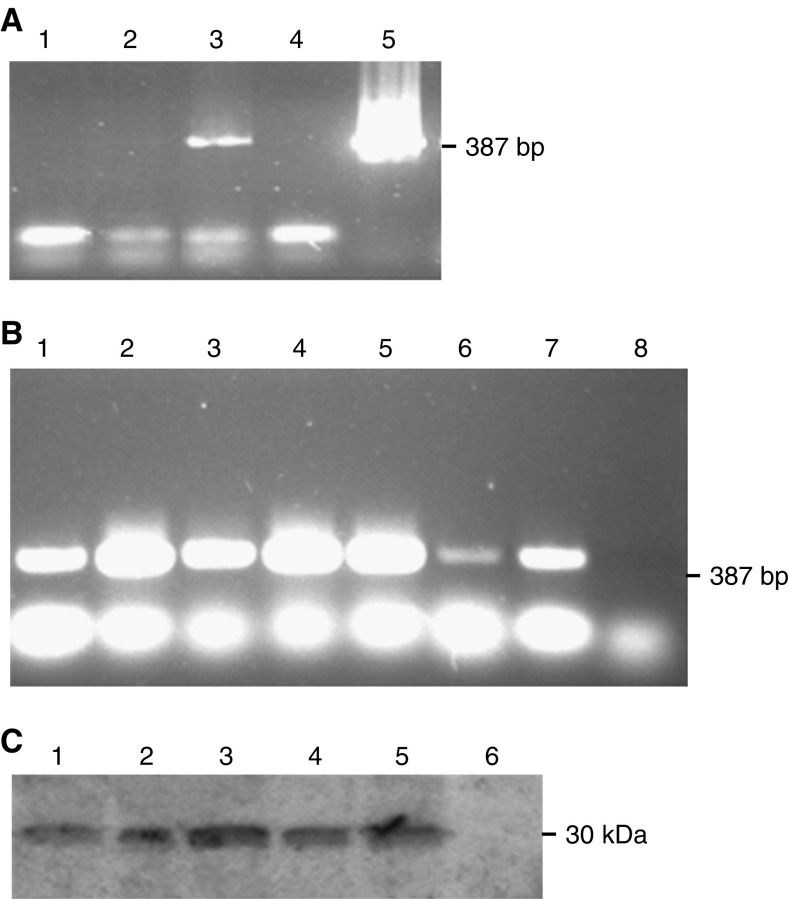
Generation of stable dermcidin transfectants. (**A**) Dermcidin PCR. Lane 1 – Untransfected HuH7 cDNA. Lane 2 – Empty vector transfected HuH7 cDNA. Lane 3 – pcDNA3.1+PIF transfected HuH7 cDNA. Lane 4 – Blank. Lane 5 – pcDNA3.1+PIF plasmid. Cells were cultured under standard conditions and stably transfected using Fugene with G418 selection. RNA was extracted using Trizol, DNAse treated, reverse transcribed and amplified by PCR using primers to the coding region of the PIF cDNA. RNA from the HuH7 cell line was positive for PIF message only after transfection with pcDNA3.1+PIF. (**B**) Dermcidin PCR. Lane 1 – Empty vector transfected HuH7 cDNA. Lane 2 – pcDNA3.1+PIF transfected HuH7 cDNA. Lane 3 – pcDNA3.1+N32Q transfected HuH7 cDNA. Lane 4 – pcDNA3.1+N44Q transfected HuH7 cDNA. Lane 5 – pcDNA3.1+N32QN44Q transfected HuH7 cDNA. Lane 6 – untransfected HuH7 cDNA. Lane 7 – Positive control (CF-PAC cDNA). Lane 8 – Negative control (no cDNA). All transfected cells were strongly positive for dermcidin expression. Untransfected cells, which had been maintained in culture for the same period of time, became weakly positive for expression. (**C**) Immunoblot for neomycin phosphotransferase from transfected cells. Lane 1 – Empty vector transfected HuH7 lysate. Lane 2 – pcDNA3.1+PIF transfected HuH7 lysate. Lane 3 – pcDNA3.1+N32Q transfected HuH7 lysate. Lane 4 – pcDNA3.1+N44Q transfected HuH7 lysate. Lane 5 – pcDNA3.1+N32QN44Q transfected HuH7 lysate. Lane 6 – untransfected HuH7 lysate. Membranes were probed with a monoclonal antibody to neomycin phosphotransferase. All transfected cells demonstrated expression from the pcDNA3.1+ CMV-driven promoter. No expression was observed in untransfected cells.

PCR demonstrated that untransfected, unstimulated HuH7 cells did not express PIF/dermcidin (Figure 4A). However, following prolonged culture, a low level of DCD expression was observed (Figure 4B). Following transfection and selection, strong expression was observed (Figure 4B). The expression observed in empty pcDNA3.1+ transfected cells raised the possibility that expression may be induced by the transfection and selection process. However, anti-neomycin phosphotransferase Western blotting confirmed the activity of pcDNA3.1+'s constitutive CMV promoter region in all transfected cells (Figure 4D). This promoter also drives the multiple cloning site. All aspects of transfection which may affect cell behaviour, except dermcidin overexpression from the vector, are subsequently controlled for.

### Analysis of HuH7 cell response to oxidative stress

**Figure 5:**
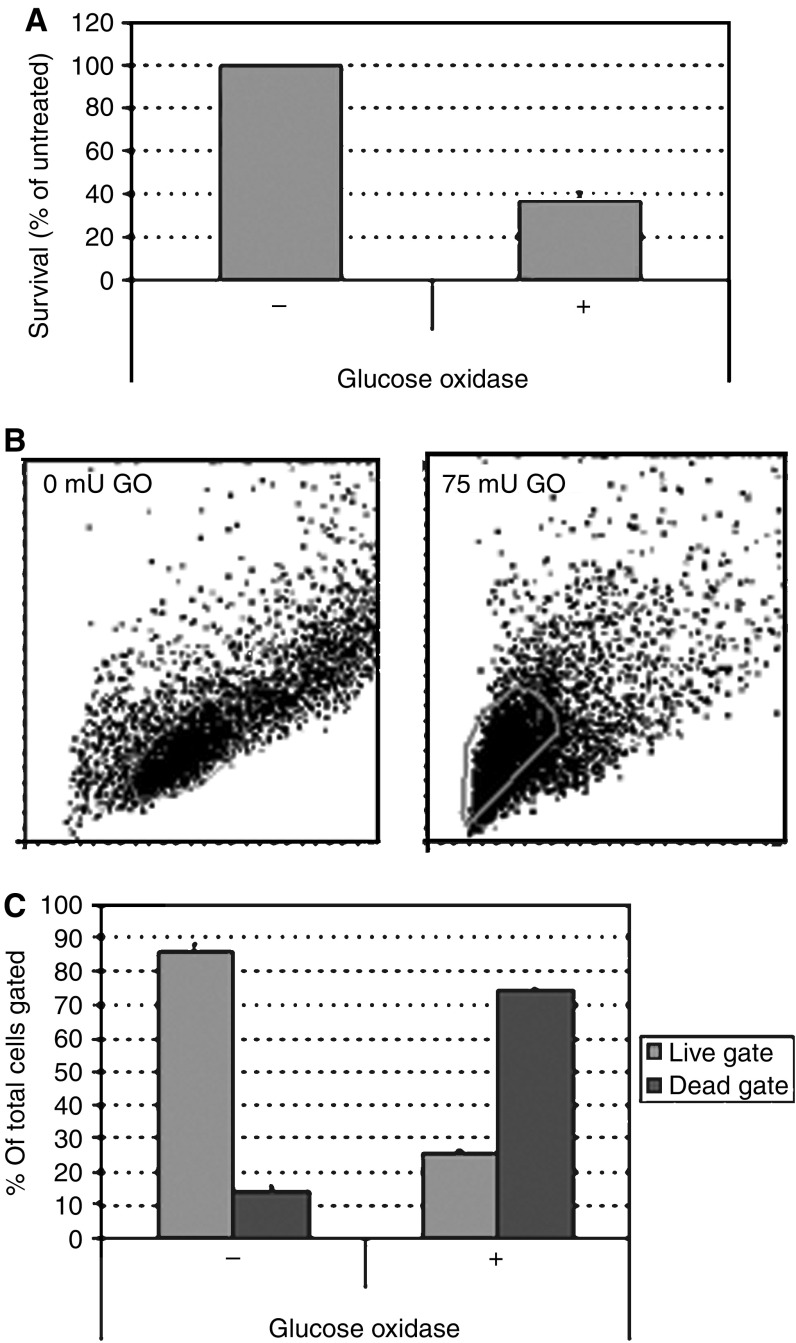
Effect of glucose oxidase on sham-transfected HuH7 cells. (**A**) MTT assay. 15 × 10^4^ cells per well were seeded in 96-well plates, incubated overnight, then treated with 75 mU ml^−1^ glucose oxidase for 2 h. MTT uptake by viable cells was assessed by the addition of 50 *μ*g MTT per well for 4 h. Plates were read at 570 nm following further overnight incubation with 100 *μ*l per well of 10% SDS, pH 3.0. Glucose oxidase treatment resulted in a reduction in the viable cell population to 38% of untreated cells. (**B**) Gating of live and dead cells by forward- and side-scatter on flow cytometry. Cells were seeded in six-well plates (1 × 10^6^ cells per well), incubated overnight, treated for 2 h with 75 mU ml^−1^ glucose oxidase, then harvested. Following two washes in PBS, flow cytometry was performed on a Coulter Epics XL flow cytometer. Treatment with glucose oxidase resulted in a marked shift in the cell population, increasing mean density and decreasing size. The resultant populations were subsequently gated as live and dead. The spread of cells into the dead gate in untreated samples was constant throughout our experiments, but not observed in untransfected HuH7s, and was considered to represent an effect of ongoing selection with G418. (**C**) Quantification of live and dead gates revealed that GO treatment resulted in a 65% shift from live to dead gates.

**Figure 6:**
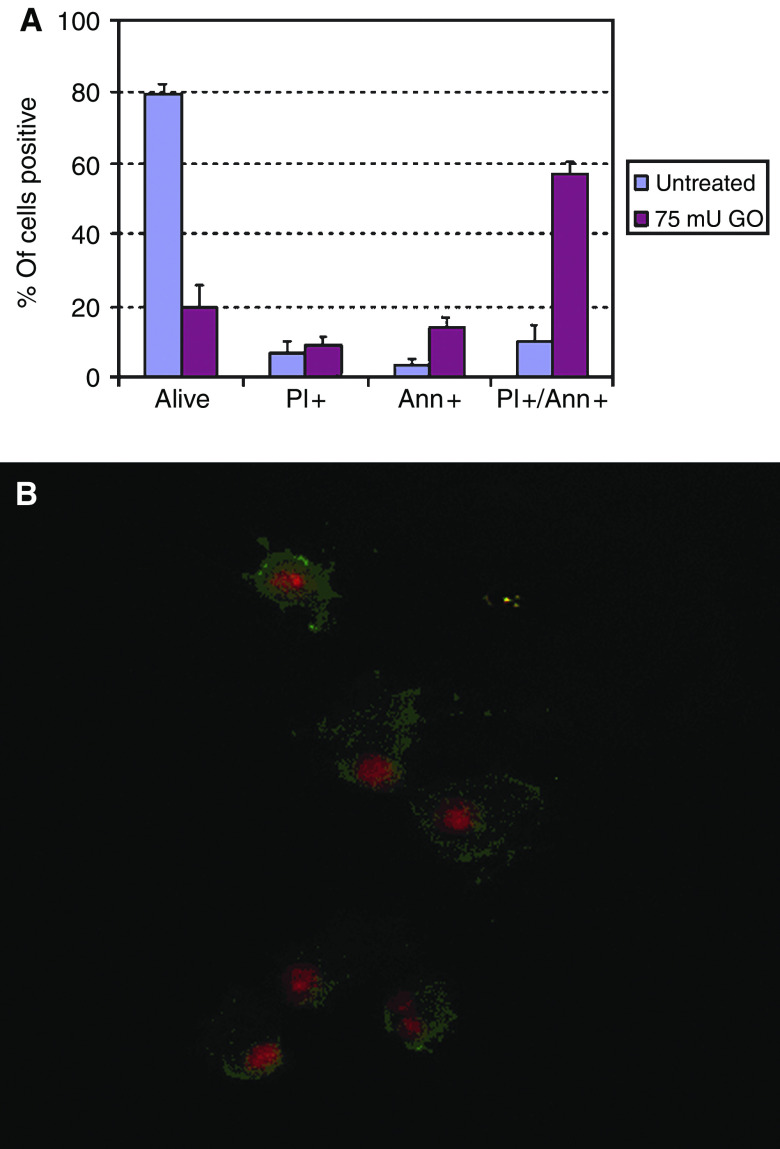
Effect of glucose oxidase on annexin V staining of HuH7 cells. (**A**) Flow cytometry. Following glucose oxidase treatment, cells were harvested, washed and stained with annexin V and propidium iodide. Glucose oxidase increased the proportion of cells staining with annexin only and with both annexin and PI. (**B**) Immunocytochemistry. Cells were seeded at 2 × 10^4^ per well in eight-well chambered slides, incubated overnight and treated for 2 h with 75 mU ml^−1^ glucose oxidase. The medium was then harvested and cytospun on to charged slides at 300 g for 3 min. Cytospins were stained with annexin V and propidium iodide and visualised using fluorescence microscopy. The majority of cytospun cells had a necrotic phenotype. However, some showed features of apoptosis including loss of cytoplasm, blebbing and nuclear condensation (top cell). Almost all cells co-stained with annexin and PI suggesting any apoptotic cells had progressed to lose membrane integrity.

**Figure 7:**
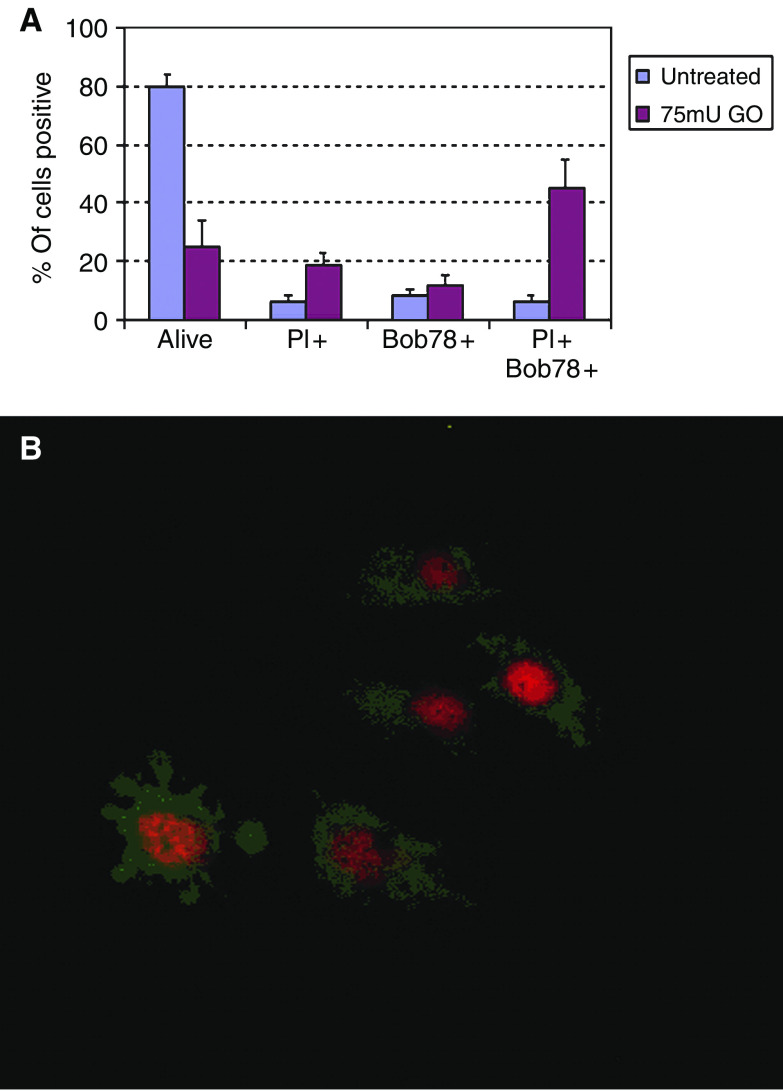
Effect of glucose oxidase on BOB78 staining of HuH7 cells. (**A**) Flow cytometry. Following glucose oxidase treatment, cells were harvested, washed and co-stained with BOB78 antibody at 1/100 in PBS and propidium iodide. Treatment with glucose oxidase resulted in a decrease in nonstaining cells and an increase in both BOB78 only and double BOB78 and PI staining. (**B**) Immunocytochemistry. Cells were seeded at 2 × 10^4^ per well in eight-well chambered slides, incubated overnight and treated for 2 h with 75 mU ml^−1^ glucose oxidase. The medium was then harvested and cytospun on to charged slides at 300 g for 3 min. Cytospins were stained with BOB78 (1/100 in PBS) and propidium iodide and visualised using fluorescence microscopy. Cytospun cells again demonstrated predominantly necrotic morphology. A few cells with apoptotic features were seen (left), but again almost all of these were PI permeable. The pattern of BOB78 staining was predominantly cytoplasmic in necrotic cells and showed some redistribution to the plasma membrane in apoptosis, consistent with our previous findings.

Empty vector-transfected cells were subjected to oxidative stress using glucose oxidase. Treatment for 2 h with 75 mU ml^−1^ glucose oxidase resulted in a decrease in survival of 66% on MTT assay (Figure 5A). On flow cytometry, forward- and side-scatter gated 82% of untreated cells as live and 18% as dead (Figure 5B and C). Glucose oxidase treatment decreased the live gate to 29%, a 65% reduction (Figure 5D). There was a commensurate increase in the dead gate to 71%. Glucose oxidase-treated HuH7 cells did not stain with anti-cleaved caspase 3 antibodies. Analysis of annexin V staining patterns revealed that untreated, 4% stained with annexin only, 7% with PI only, 11% with both and 79% with neither (Figure 6A). When treated with glucose oxidase, 14% stained with annexin only, 9% with PI only, 58% with both and 19% with neither. On staining of cells with the BOB78 antibody, a surface marker of apoptosis (Hart *et al*, 2000), 8% stained with BOB78 only in the untreated sample, 6% with PI only, 6% with both and 80% with neither (Figure 7A). On glucose oxidase treatment, 12% stained with BOB78 only, 18% with PI only, 45% with both and 253% with neither (Figure 7A). Immunocytochemistry with both annexin V and BOB78 (Figures 6B and 7B) also revealed a predominantly necrotic morphology and staining pattern. Glucose oxidase therefore appears to induce necrosis in HuH7 cells.

### Effect of PIF/dermcidin expression on cell survival

**Figure 8:**
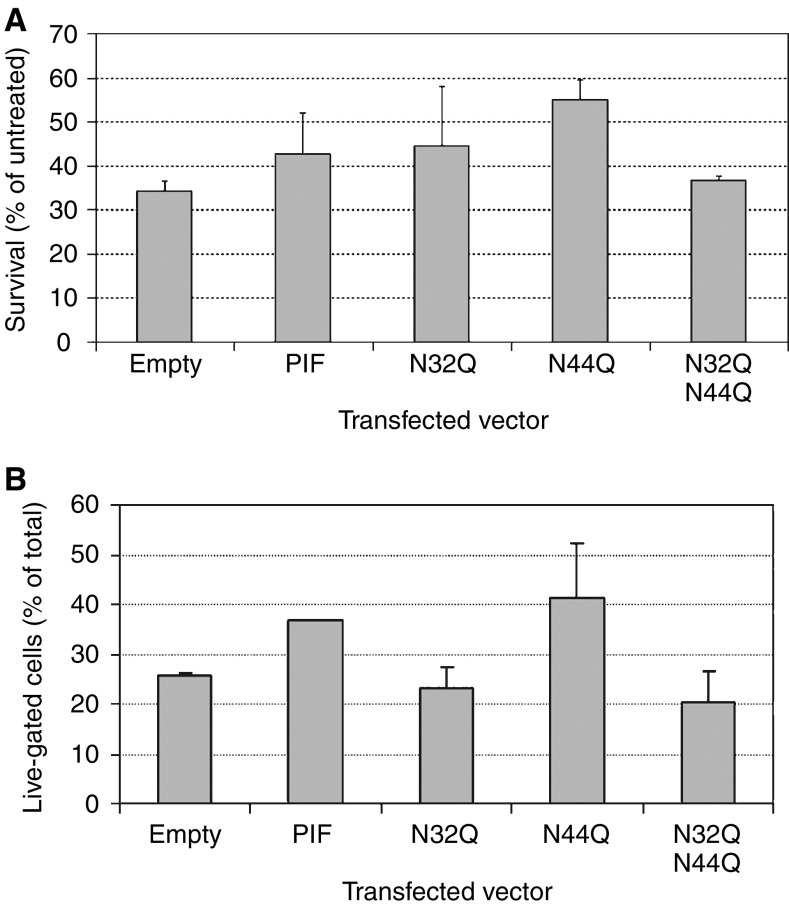
Effect of transfected vector on survival after oxidative stress. (**A**) MTT assay. Cells transfected with empty vector (sham transfectants), wild-type PIF or the N32Q, N44Q or N32Q N44Q mutant forms of PIF were seeded at 15 × 10^4^ cells per well in 96-well plates, incubated overnight, then treated with 75 mU ml^−1^ glucose oxidase for 2 h prior to MTT assay. Wild-type PIF transfection resulted in a 26% increase in viable cells following glucose oxidase treatment in comparison with sham transfection. Transfection with the N32Q and N44Q mutants similarly protected HuH7 cells from oxidative stress. In contrast, transfection with the double mutant N32Q N44Q did not bestow a survival benefit. (**B**) Flow cytometry.Transfected cells were seeded at 1 × 10^6^ cells per well in six-well plates, incubated overnight, treated for 2 h with 75 mU ml^−1^ glucose oxidase, then harvested. Flow-cytometric gating of cells by forward and side scatter revealed 37% survival of wild-type PIF transfected cells in comparison with 26% survival of sham transfectants, a survival benefit of 42%. Transfection with the N44Q mutant bestowed a similar benefit to transfection with wild-type PIF. Neither the N32Q nor the N32Q N44Q mutants conferred any survival benefit.

**Figure 9:**
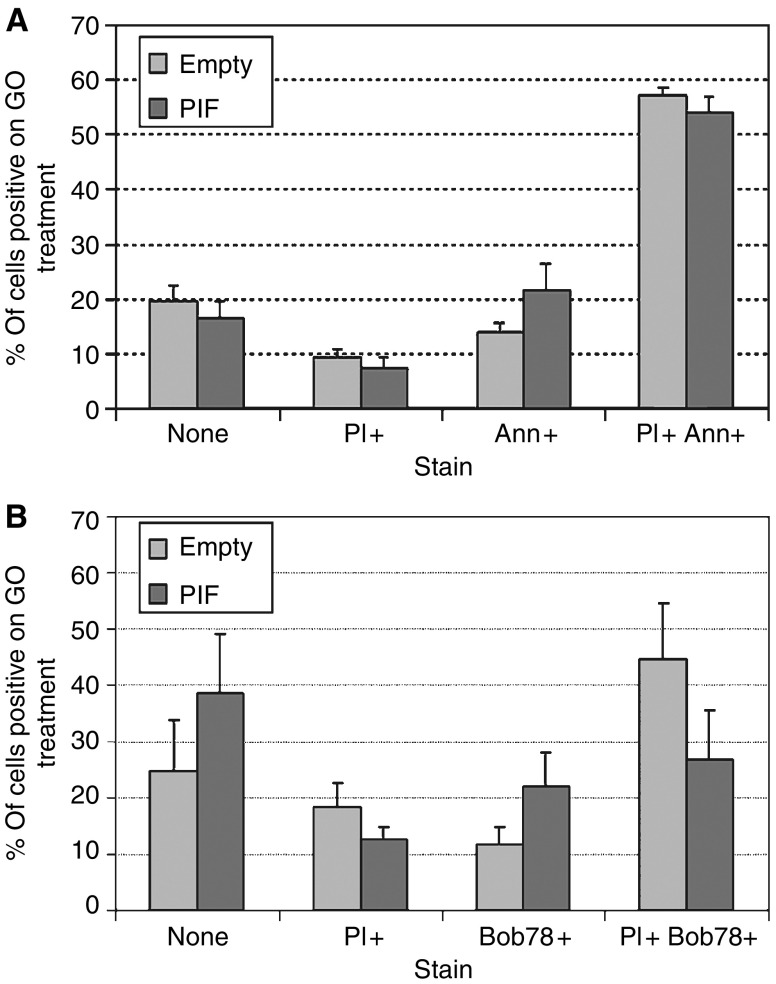
Effect of transfection with wild-type PIF/dermcidin on flow-cytometric staining patterns of glucose oxidase-treated cells. (**A**) Annexin V. Cells were seeded at 1 × 10^6^ per well, treated with glucose oxidase, harvested, and stained with annexin V and propidium iodide as before. Proteolysis-inducing factor transfection resulted in a change in annexinV only staining from 15 to 22%, PI only staining from 9 to 8%, double staining from 57 to 54% and no staining from 19 to 16%. (**B**) BOB78. Cells were seeded at 1 × 10^6^ per well, treated with glucose oxidase, harvested, and stained with BOB78 (1/100 in PBS) and propidium iodide as before. The proportion of cells staining with BOB78 only increased from 12 to 22% on PIF transfection. BOB78 and PI double staining decreased from 45 to 27%. Nonstaining cells increased from 25 to 39% and PI only staining changed from 19 to 13%.

Following glucose oxidase treatment, the mean survival of HuH7 cells as assessed by MTT assay was increased from 34 to 43% by transfection of the PIF/dermcidin-containing vector, a survival benefit of 26% (Figure 8A). On flow cytometry, forward- and side-scatter gating demonstrated an increase in live-gated cells from 26 to 37% following PIF/dermcidin transfection, a survival benefit of 42% (Figure 8B). Proteolysis-inducing factor/dermcidin-transfected HuH7 cells did not stain with anti-cleaved caspase 3 antibody. Analysis of the annexin V staining pattern of PIF/dermcidin-transfected cells demonstrated an increase in the number of cells staining with annexin only and a decrease in the number staining with annexin V and PI (Figure 9A). Analysis of the BOB78 staining pattern of PIF/dermcidin-transfected cells revealed an increase in the number of cells staining with BOB78 alone and with neither BOB78 nor PI, and a decrease in the number of cells staining with both BOB78 and PI (Figure 9B). These results suggest that dermcidin transfection improves cell survival by decreasing necrosis, with an increase in both live cells and cells undergoing apoptosis.

### Effect of site-directed mutagenesis of asparagine residues

We substituted glutamine for each of the two asparagine residues in the PIF/dermcidin polypeptide sequence, both singly and in combination. When transfected into HuH7 cells, the single mutations N32Q and N44Q conferred the same survival benefit as wild-type PIF/dermcidin, as assessed by MTT assay (Figure 8A). However, on flow cytometry, the N32Q mutation abrogated the effect on cell survival, whereas the N44Q mutation had no effect (Figure 8B). Double mutation of N32 and N44 removed the ability of PIF/dermcidin expression to protect cells as assessed by both MTT assay and flow cytometry (Figure 8A and B). No significant difference in the annexin V and BOB78 staining patterns between N32Q, N44Q and wild-type PIF/dermcidin or N32Q N44Q and sham transfectants was observed.

## DISCUSSION

Previous studies have shown that glucose oxidase induces necrosis in hepatic cells through the constant generation of H_2_O_2_ and subsequently free oxygen radicals (Lemasters, 1999). In HuH7 cells we found that cell death occurred almost exclusively by necrosis. In particular, there was no activation of cleaved caspase 3, a mediator common to both the receptor-activated and intrinsic pathways of apoptosis, which is known to be functional in HuH7 cells (Ashkenazi and Dixit, 1998; Feng and Kaplowitz, 2002). The low level of annexin staining in comparison with double annexin/PI staining on both flow cytometry and immunocytochemistry was also consistent with necrotic cell death. In addition, BOB78 expression showed a similar pattern of change to annexin V.

Using the glucose oxidase system, we demonstrated a clear survival benefit of PIF/dermcidin expression. On MTT assay this was 26% and on forward- and side-scatter gated flow cytometry, 25%. Shifts from cell apoptosis to necrosis are known to occur in primary hepatocytes, where the inhibition of caspase activity converts apoptotic to necrotic cell death in the presence of an overwhelming death stimulus from staurosporine (Feng and Kaplowitz, 2002). However, this did not occur in the HuH7 cells, as cleaved caspase 3 was not activated. In addition, with dermcidin transfection, the main changes in annexin V staining were a rise in annexin only and a fall in double annexin/PI staining. This represents a proportional decrease in necrosis on PIF/dermcidin expression when considered in light of the MTT assay and forward- and side-scatter results, which demonstrated that the population of dead cells is smaller in dermcidin-transfected cells. The implications of these findings are potentially wide-ranging. For example, it is possible that during hepatic carcinogenesis dermcidin expression could improve the ability of cells to survive in areas of high oxidative stress, resulting in the positive selection of cells with a survival advantage. Preliminary results suggest that certain hepatic carcinoma cell lines express dermcidin, and the development of siRNAs will facilitate investigation of the effects of inhibiting expression in these cells and in overexpressing HuH7s. Demonstration of altered expression in hepatic tumours would confirm the potential of dermcidin to act as an oncogene in tumours other than breast cancer. Our findings also suggest that dermcidin could be used to pre-condition nonmalignant hepatic cells to oxidative stress in a manner similar to other therapies such as cyclosporin and heat shock (Lemasters *et al*, 1998). If dermcidin does play a role in the hepatic response to oxidative stress, its pattern of expression might be expected to be pericentral, where hepatocyte cytochrome *p*450 epxression is high and oxygen tensions are low, resulting in oxidative stress. The development of immunohistochemistry and *in situ* hybridisation will help to investigate patterns of expression in *in vivo* models.

As glycosylation appears essential to the ability of PIF to induce cachexia, we attempted to correlate the ability of dermcidin expression to promote survival with structural mutations designed to alter *N*-glycosylation. This revealed that, although the N44Q mutation had no effect on survival assessed by either MTT assay or flow cytometry, the N32Q mutation prevented dermcidin's pro-survival effects when assessed by flow cytometry, but not by MTT assay. As the MTT assay measures cell survival indirectly by assessing mitochondrial function, this difference suggests that the N32 residue may mediate a pro-survival effect independent of mitochondrial function. Dermcidin may subsequently influence survival via two or more mechanisms. Preconditioning to oxidative stress by enzymatic induction is well described in hepatic cells, is active in neuronal cells (in which Y-P30 is protective) (Arthur *et al*, 2004), and may be one mechanism by which dermcidin acts in HuH7 cells. Alternatively, expression might protect cells from oxidative stress by induction of the proteasome system, which is known to be activated by glycosylated PIF (Smith and Tisdale, 2003; Whitehouse and Tisdale, 2003). Proteasome induction has been associated with improved survival of both neuronal cells and liver cells following oxidative stress (Donohue Jr *et al*, 2004; Lee *et al*, 2004). Finally, the fact that double mutation of N32 and N44 contrasted with single N32 mutation in abrogating the survival effect on MTT assay suggests that a mitochondrial pathway may also be involved. The mitochondrial permeability transition may be induced by oxidative stress and is increasingly recognised as an element of cell necrosis (Lemasters *et al*, 1998; Lemasters, 1999). This transition may be blocked by cyclosporin A, an inhibitor of calcineurin, with which dermcidin shares a homologous phosphatase domain (Cunningham *et al*, 1998; Rusnak and Mertz, 2000), raising the possibility that this may be the point at which dermcidin acts on the necrotic pathway.

The structural effects of the asparagine residues which influence the function of dermcidin have not been determined in the present study. Several possibilities exist. The motif identified in Y-P30 as similar to the calcineurin phosphatase domain lies between N32 and N44, and has been demonstrated to have phosphatase activity (Cunningham *et al*, 1998, 2000). X-ray crystallography has shown that asparagine residues contribute to this domain in calcineurin (Rusnak and Mertz, 2000). It is therefore feasible that loss of both asparagine residues but not one alone is sufficient to impair such phosphatase activity. Alternatively, the asparagine residues may be important in receptor binding, proteolytic processing, or protein folding or integrity. The receptors for the different products of dermcidin remain largely uncharacterised, although both high- and low-affinity receptors on breast cancer cells bind dermcidin with alkaline phospatase attached to the DCD-1 carboxy-terminus (Porter *et al*, 2003) and glycosylated PIF has been suggested to bind to both hepatocytes and skeletal muscle (Waddell *et al*, unpublished observation). Y-P30 has been demonstrated to interact with calreticulin (Cunningham *et al*, 2000), although it is not known whether this also interacts with PIF or DCD-1. It is therefore possible that asparagine mutagenesis in such a short sequence might affect dermcidin's receptor interactions. As polar amino acids, the hydrophobicity of both asparagine and glutamine is similarly low (Nozaki and Tanford, 1971) and both have similar structures. This has resulted in the common use of glutamine for asparagine substitutions in experiments examining *N*-glycosylation, where as little effect as possible on other aspects of protein structure is desirable. However, due to their low hydrophobicity, both amino acids are commonly found on the surface of globular proteins and mutagenesis could therefore have an effect on the conformation of the short dermcidin peptide, with subsequent effects on receptor binding or other biological activities. It is also possible that the substitution may alter proteolytic processing by rendering a protease target site either more or less accessible. A subsequent change in the ratios of the different dermcidin products produced could be expected to alter the biological effect of expression.

We were unable to demonstrate evidence of *N*-glycosylation in the rabbit-reticulocyte lysate *in vitro* translation system, where elimination of one or both asparagine residues failed to alter the molecular weight of translated dermcidin. This contrasts with previous studies, which have suggested heavy *N*-glycosylation of up to 10 kDa, possibly at one site in the 24 kDa moiety (Todorov *et al*, 1997), but is consistent with the observation that neither site represents a typical consensus *N*-glycosylation sequence, although N32 has the characteristics of an atypical site (Welply *et al*, 1983; Sato *et al*, 2000). The *in vitro* translation system was used to assess dermcidin *N*-glycosylation, as its immunological analysis is problematic. The small size of the peptide and its proteolytic products results in limited antigens to which antibodies may be developed. Previous studies have used antibodies recognising epitopes that are glycosylated (Todorov *et al*, 1996) or involve the mutated asparagines (Cunningham *et al*, 1998). However, the use of radiolabelling in the *in vitro* translation system avoids these difficulties and it is well recognised as a model of mammalian *N*-glycosylation. The lack of *N*-glycosylation therefore suggests that it is not involved in the mediation of survival effects through dermcidin's asparagine residues. However, the fact that the system may not accurately represent glycosylation in HuH7 cells must be considered.

Whether dermcidin is targeted to the secretory pathway has been difficult to determine from previous literature. Several *in vitro* studies of PIF have used immunological methods of detection and have always required cell lysates, as opposed to supernatants, for success (Todorov *et al*, 1996, 1999). In contrast with these findings, *in vivo* studies using the same antibody have demonstrated glycosylated PIF in urine and suggest that it may be found, bound to albumin, in serum, suggesting that the molecule may be secreted (Todorov *et al*, 1996; Wigmore *et al*, 2000). The DCD-1 and Y-P30 peptides are secreted, having been identified in sweat and cell culture medium, respectively (Cunningham *et al*, 1998; Schittek *et al*, 2001). The apparent conflict between this evidence and the previous *in vitro* PIF studies may be accounted for by the use of different antibodies to detect Y-P30, DCD-1 and PIF. The anti-PIF antibody has been shown to recognise a carbohydrate epitope (Todorov *et al*, 1997) and it is feasible that, when glycosylated, the peptide is not secreted and requires cell lysis for release. We have demonstrated that the dermcidin signal peptide is cleaved and that inhibition of protein transport is required to detect dermcidin in HuH7s, supporting secretion in these cells. However, as the V5-His tag was attached to the carboxy-terminus, if dermcidin is proteolytically processed, only DCD-1 may be labelled and therefore we cannot comment on whether Y-P30 or PIF are secreted or even produced by these cells. Further studies of the antigen specificity of the different anti-PIF/dermcidin antibodies and careful analysis of glycosylation in different cell types will be required to address these issues using immunological methods.

Our results complete the cloning of PIF, Y-P30 and dermcidin, confirming that they arise from the same polypeptide, cDNA, and gene, dermcidin. No other sequence in the human genome shows homology on BLAST searching. Why such diverse functions should have evolved in products of the same gene is unclear. Proteolysis-inducing factor was originally purified from a murine tumour (MAC 16) and would therefore be expected to have a murine homologue. However, although partial synteny between the coding sequence for the N-terminus of PIF and a sequence on murine chromosome 15 has been found, a full match does not appear to exist. This may reflect strain variations in the mouse genome database, which was developed from the C57BL/6J strain. Additional sequences with homology to the dermcidin sequence are present in the genome of the chimpanzee *Pan troglodytes, Rattus norvegicus* and *caenorhabditis elegans* (genbank accession numbers NM_173108/AF531422 and AC024770, respectively), but have as yet to be located in the zebrafish genome. The PIF/dermcidin coding sequence therefore appears to demonstrate evolutionary conservation, suggesting that the gene may have an important cellular function. Several features of the dermidin cDNA suggest that production of its various products may be regulated at the post-transcriptional or post-translational levels. Firstly, there are no alternative start sites in the sequence. Secondly, the sequence of Y-P30 can be directly deduced from continuous exons and the cleavage site of the signal peptide is encoded entirely by exon 1, suggesting that they are not products of differential splicing. It is therefore likely that differential proteolysis is responsible for the production of the different dermcidin peptides and in sweat, four different peptides have been identified, which appear to be proteolytic products (Flad *et al*, 2002). The specific proteases involved remain to be determined. Thirdly, PIF shares the same core peptide sequence as Y-P30, but is highly glycosylated (Todorov *et al*, 1997).

We have demonstrated that the survival effect of dermcidin, potentially mediated via the calcineurin-like phosphatase domain, is active in cells known to respond to glycosylated PIF. Post-translational modification is almost certainly required for the cachexia-inducing effects demonstrated by Todorov *et al* (1996) and the abnormal glycosylation known to occur in neoplasia may prove to be the mechanism by which this occurs. Further studies of expression, structure and post-translational modification have great potential to characterise further the functions of products of this gene.
